# Naturally acquired antibody responses to more than 300 *Plasmodium vivax* proteins in three geographic regions

**DOI:** 10.1371/journal.pntd.0005888

**Published:** 2017-09-11

**Authors:** Rhea J. Longley, Michael T. White, Eizo Takashima, Masayuki Morita, Bernard N. Kanoi, Connie S. N. Li Wai Suen, Inoni Betuela, Andrea Kuehn, Piyarat Sripoorote, Camila T. Franca, Peter Siba, Leanne J. Robinson, Marcus Lacerda, Jetsumon Sattabongkot, Takafumi Tsuboi, Ivo Mueller

**Affiliations:** 1 Population Health and Immunity Division, Walter and Eliza Hall Institute of Medical Research, Melbourne, Victoria, Australia; 2 Department of Medical Biology, University of Melbourne, Melbourne, Australia; 3 Mahidol Vivax Research Unit, Faculty of Tropical Medicine, Mahidol University, Bangkok, Thailand; 4 MRC Centre for Outbreak Analysis and Modelling, Department of Infectious Disease Epidemiology, Imperial College London, London, United Kingdom; 5 Division of Malaria Research, Proteo-Science Center, Ehime University, Matsuyama, Japan; 6 Vector Borne Diseases Unit, PNG Institute of Medical Research, Madang, Papua New Guinea; 7 ISGlobal, Barcelona Centre for International Health Research (CRESIB), Hospital Clinic-Universitat de Barcelona, Barcelona, Spain; 8 Fundação de Medicina Tropical Dr. Heitor Vieira Dourado, Manaus, Brazil; 9 Instituto de Pesquisas Leônidas e Maria Deane, Manaus, Amazonas, Brazil; 10 Malaria: Parasites & Hosts Unit, Department of Parasites & Insect Vectors, Institut Pasteur, Paris, France; Johns Hopkins Bloomberg School of Public Health, UNITED STATES

## Abstract

*Plasmodium vivax* remains an important cause of malaria in South America and the Asia-Pacific. Naturally acquired antibody responses against multiple *P*. *vivax* proteins have been described in numerous countries, however, direct comparison of these responses has been difficult with different methodologies employed. We measured antibody responses against 307 *P*. *vivax* proteins at the time of *P*. *vivax* infection, and at 2–3 later time-points in three countries. We observed that seropositivity rates at the time of infection were highest in Thailand, followed by Brazil then PNG, reflecting the level of antigenic input. The majority of sero-reactive antigens in all sites induced short-lived antibody responses with estimated half-lives of less than 6 months, although there was a trend towards longer-lived responses in PNG children. Despite these differences, IgG seropositivity rates, magnitude and longevity were highly and significantly rank-correlated between the different regions, suggesting such features are reflective of the individual protein.

## Author summary

In the pursuit of eliminating all species of malaria, *Plasmodium vivax* presents one of the most substantial challenges, particularly in countries in Asia, the Western-Pacific and South America. This is primarily due to the ability of *P*. *vivax* to cause relapse infections months to years after the initial infectious bite. In areas with low levels of malaria transmission, serology has become an increasingly useful tool for surveillance, as anti-*Plasmodium* antibodies can be detected in individuals long after blood-stage parasites have cleared. In this study, we provide a detailed characterisation of the antibody response generated following *P*. *vivax* infection by measuring antibodies to over 300 *P*. *vivax* antigens in three different populations in Thailand, Brazil and Papua New Guinea. The individuals in these populations were followed for up to nine months allowing us to estimate the rate at which antibodies decay over time. This improved understanding of the magnitude and dynamics of the antibody response, validated in multiple populations, will contribute to the development of serological surveillance tools needed for enhanced control and elimination of *P*. *vivax*.

## Introduction

*Plasmodium vivax* is the most geographically widespread of the *Plasmodium* spp. causing malaria in humans, and accounts for the majority of cases outside Africa [1]. The leaders of Central American and East Asian countries have declared their intention for elimination of malaria within their regions by 2025 and 2030 [2, 3], respectively. Interrupting *P*. vivax transmission may require the development of an effective vaccine and improved surveillance systems (due to the high proportion of sub-microscopic *P*. *vivax* infections [4] and the presence of currently undetectable hypnozoites) [5]. Both objectives require a better understanding of naturally induced immune responses, in particular antibody responses, following infection.

Protective immunity against clinical episodes of *P*. *vivax* malaria is acquired more rapidly than immunity against *P*. *falciparum* [6, 7]. As a consequence, the burden of clinical *P*. *vivax* disease falls predominantly on very young children, whilst infected adults are often asymptomatic [8–10]. Immunity against clinical symptoms is thought to be dependent on the development of *P*. *vivax*-specific antibody responses [11, 12]. The prevalence of *P*. *vivax-*specific antibody responses within endemic populations increases with age [13], and antibody levels are generally higher in individuals with current *P*. *vivax* infections [14]. The parasite expresses more than 5000 proteins throughout its lifecycle [15], and through the use of large-scale screening platforms such as protein microarrays [16], an increasing number of these proteins have been assessed for their immunogenicity in naturally exposed populations. These studies, assessing antibody responses to over 1900 *P*. *vivax* proteins, have indicated that up to 50% of such proteins are sero-reactive in individuals from malaria endemic areas [11]. Furthermore, they have demonstrated that asymptomatic individuals have a greater breadth of response [11], that reactive proteins are more likely to be encoded by genes with high single nucleotide polymorphism diversity (potentially signifying positive immune selection) [17], and that the majority of highly reactive proteins have predicted transmembrane domains and signal peptides (SP) (signifying secreted or membrane-bound proteins) [18].

Whilst naturally acquired antibody responses have been assessed against multiple *P*. *vivax* proteins in individuals from numerous geographic regions, it has been difficult to directly compare these responses due to the different protein expression and antibody measurement systems used. For example, whilst Finney and colleagues reported a greater breadth of antibody response in asymptomatic individuals in PNG compared to febrile patients [11], as noted above, the opposite was later reported in Thailand [13] and no difference was identified in India [19]. This could be due to the different geographic region and transmission intensity of the study sites, or due to the use of different *P*. *vivax* protein microarrays encompassing different sets of proteins.

In this study we performed a direct comparison of naturally acquired antibody responses in three different geographic regions: Brazil (very low transmission), Thailand (low transmission) and PNG (moderate transmission). In these settings, we measured antibodies at 2–3 time-points following *P*. *vivax* infection (in the absence of detectable recurrent infections), to a panel of 307 *P*. *vivax* proteins. All samples were measured using the same platform (AlphaScreen assay), with the same data analysis pipeline applied. We found that antibody positivity, magnitude and longevity were highly and significantly correlated between the three study sites; however, in general, antibody longevity was better maintained in PNG where individuals have had greater past exposure to the parasite. Our study provides important new insights into the generation and maintenance of *P*. *vivax*-specific antibody responses and how these factors differ in regions of varying transmission intensity.

## Methods

### Ethics statement

The relevant local ethics committees approved all field studies, and the Human Research Ethics Committee at WEHI approved samples for use in Melbourne (#14/02). The PNG study received ethical clearance from the PNG Institute of Medical Research Institutional Review Board (0908), the PNG Medical Advisory Committee (09.11), and the Ethics Committee of Basel (237/11). This study was also retrospectively registered at ClinicalTrials.gov (NCT02143934). The Thai study was approved by the Ethics Committee of the Faculty of Tropical Medicine, Mahidol University, Thailand (MUTM 2014-025-01 and 02). The Brazilian study was approved by the Ethics Review Board of the Fundação de Medicina Tropical Dr. Heitor Vieira Dourado (FMT-HVD) (957.875/2014). All patients gave informed written consent or assent. For children, informed written consent was provided by their parents.

### Study sites

Three different study sites were used: Maprik District, East Sepik Province, Papua New Guinea (PNG); Tha Song Yang District, Tak Province, Thailand; and Manaus, Brazil. A sub-set of enrolled volunteers, where the probability of reinfection during the study period was low (in order to accurately determine antibody longevity), was included in the present study as described below.

The longitudinal study in PNG was conducted from August 2009 to May 2010, as described [20]. In brief, 524 children aged 5–10 years were enrolled and block randomised to receive 20 days of directly observed therapy (DOT) of chloroquine, arthemeter-lumefrantrine with either a placebo or primaquine. Children were subsequently monitored every 2 weeks for 8 months to detect any signs or symptoms of infection, with finger-prick blood samples taken every 2 weeks for 12 weeks, then every 4 weeks for the remaining period. Blood samples were analysed by light microscopy and qPCR for the presence of blood-stage parasites and plasma stored for antibody measurements. A sub-set of 31 children with *P*. *vivax* infections at enrolment and no evidence of reinfection during follow-up, with not more than one missed sample, were selected for inclusion in the present study (all 31 received primaquine treatment). Plasma samples from 0, 3 and 5 months were used.

The longitudinal study in Thailand was conducted from April 2014 to September 2015, as described [21]. Briefly, 57 symptomatic *P*. *vivax* patients were enrolled from either the Tha Song Yang malaria clinic or hospital. Patients were treated with chloroquine (25 mg base/kg body weight, administered over 3 days) and primaquine (15 mg daily, for 14 days) under DOT. Volunteers were followed for 9-months after enrolment, with finger-prick blood samples collected at enrolment and week 1, then every 2 weeks for 6 months, then every month until the end of the study. All blood samples were analysed by both light microscopy and qPCR for the presence of blood-stage parasites as per the PNG cohort, and plasma separated and stored. A sub-set of volunteers, n = 32, were selected for use in the current study. These volunteers had no detectable recurrent infections during 9-months follow-up, and were the first to complete follow-up. Plasma samples from 0, 3, 6 and 9 months were used.

The longitudinal study in Brazil followed the same format as in Thailand. The study was conducted from May 2014 to May 2015. 91 malaria patients at Fundação de Medicina Tropical Doutor Heitor Vieira Dourado in Manaus aged between 7 and 70 years were enrolled. Individuals with G6PD deficiency or chronic diseases were not enrolled. Patients were treated according to the guidelines of the Brazilian Ministry of Health (3 days chloroquine, 7 days primaquine). Follow-up intervals with finger-prick blood sample collection were as in the Thai study. A sub-set of volunteers, n = 33, who had no detectable recurrent infections during 9-months follow-up, were selected for use in the antigen discovery project. Plasma samples from 0, 3, 6 and 9 months were used.

### Antigen selection

**Fig 1:**
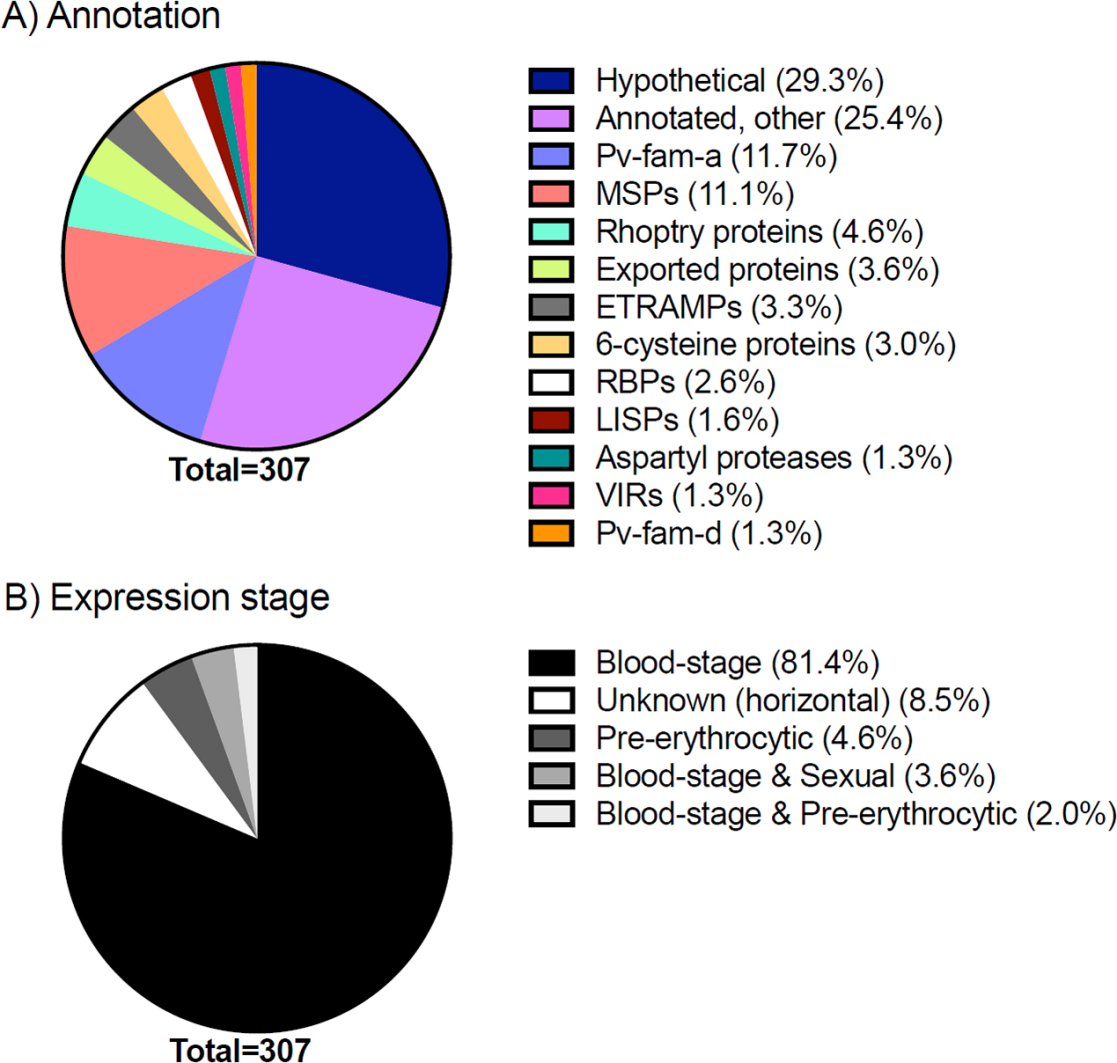
Characteristics of the 307 *P*. *vivax* proteins in the AlphaScreen assay. (A) Annotation was determined by PlasmoDB release 25. MSPs: merozoite surface proteins or paralogs, ETRAMPs: early transcribed membrane proteins, RBPs: reticulocyte binding proteins or precursors, LISPs: liver-specific proteins, VIRs: variable surface proteins. (B) Expression stage was also determined by PlasmoDB release 25.

This study utilized a panel of 307 *P*. *vivax* protein fragments produced using a wheat germ cell-free protein expression (WGCF) system. This panel of protein fragments includes well-known *P*. *vivax* proteins such as potential vaccine candidates (i.e. merozoite surface protein 1 (MSP1), apical membrane antigen 1 (AMA1), circumsporozoite protein (CSP)), orthologs of immunogenic *P*. *falciparum* proteins and proteins with a predicted SP and/or 1–3 transmembrane domains (TM), and expands upon our previously published panel [22]. Fig 1 demonstrates key features of these *P*. *vivax* proteins (annotation and expression-stage). Approximately 70% contained a predicted SP, 50% at least one TM and 10% a GPI-anchor. All protein-specific information was obtained from the Plasmodium Genomics Resource (PlasmoDB: http://plasmodb.org/plasmo/) release 25.

### WGCF protein synthesis

The recombinant proteins were expressed without codon optimization using the WGCF system as previously described [23] with slight modifications. Briefly, the genes were amplified by PCR and cloned into the pEU_E01 expression vector with N-terminal His-bls tag (CellFree Sciences, Matsuyama, Japan). *P*. *vivax* genes were obtained either from parent clones [22], using SAL-1 cDNA, or commercially synthesized from Genscript (Japan). DNA template for WGCF was prepared from the plasmids by PCR with a forward primer (spu; 5-GCGTAGCATTTAGGTGACACT-3), and a reverse primer (pbsa1143; 5-GCTCACATGTTCTTTCCTGC-3). WGCF synthesis of the *P*. *vivax* protein library was based on the previously described bilayer diffusion system [24]. For biotinylation of proteins, 500 nM D-biotin (Nacalai Tesque, Kyoto, Japan) was added to both the translation and substrate layers. Crude WGCF expressed BirA (1 μl) was added to the translation layer. In vitro transcription and cell-free protein synthesis for the *P*. *vivax* protein library were carried out using the GenDecoder 1000 robotic synthesizer (CellFree Sciences) [25, 26]. Based on previous studies, the WGCF system can synthesize biotinylated plasmodial protein ranging from 0.3 μg/mL to 26.5 μg/mL [27,28]. Expression of the proteins was confirmed by western blotting using HRP-conjugated streptavidin. Proteins were preferably expressed as full-length proteins, to ensure that any possible antibody recognition site was covered; however, for very large proteins, multiple fragments were expressed that together cover the entire protein. The panel included 263 unique proteins.

### AlphaScreen assay

The AlphaScreen assay was performed following the manufacturer’s instructions (PerkinElmer Life and Analytical Sciences, Boston, MA) as previously reported [27,28], with slight modifications. The protocol was automated by use of the JANUS Automated Workstation (PerkinElmer Life and Analytical Science, Boston, MA). Reactions were carried out in 25 μl of reaction volume per well in 384-well OptiPlate microtiter plates (PerkinElmer). First, 0.1 μl of the translation mixture containing a recombinant *P*. *vivax* biotinylated protein was diluted 50-fold (5 μl), mixed with 10 μl of 4000-fold diluted plasma in reaction buffer (100 mM Tris-HCL [pH 8.0], 0.01% [v/v] Tween-20 and 0.1% [w/v] bovine serum albumin), and incubated for 30 min at 26°C to form an antigen-antibody complex. Subsequently, a 10 μl suspension of streptavidin-coated donor-beads and acceptor-beads (PerkinElmer) conjugated with protein G (Thermo Scientific, Waltham, MA) in the reaction buffer was added to a final concentration of 12 μg/ml of both beads. The translation mixture was diluted 250 times, thus final concentration of biotinylated proteins ranged between approximately 1.2 ng/mL and 106 ng/mL. The mixture was incubated at 26°C for one hour in the dark to allow the donor and acceptor-beads to optimally bind to biotin and human IgG, respectively. Upon illumination of this complex, a luminescence signal at 620 nm was detected by the EnVision plate reader (PerkinElmer) and the result was expressed as AlphaScreen counts. Each assay plate contained a standard curve of total biotinylated rabbit IgG. This enabled standardisation between plates using a 5-paramater logistic standard curve. All samples from Thailand and Brazil were run in triplicate, whilst those from PNG were run only once. Reading the plates was conducted in a randomized manner to avoid biases.

The immunogenicity of each protein was assessed in two ways. Firstly by calculating the geometric mean titre (GMT), and secondly through seropositivity–the proportion of samples above a given threshold. The seropositivity cut-off was set at the limit of detection of the assay, taken as half the lowest non-negative value from each of the three sites [24]. Proteins were defined as reactive if more than 10% of the volunteers had levels above the seropositivity cut-off at the baseline measurement (time of *P*. *vivax* infection).

### Statistical modelling and analysis

All data manipulation and statistical analyses were performed in R version 3.2.3 [29]. Antibody longevity was estimated using data from three or four time-points (0, 3 and 5 months in PNG; 0, 3, 6 and 9 months in Thailand and Brazil). A linear mixed effects model was used to estimate the antibody half-life; using the lme4 [30] package in R. We assume that antibody titres decay exponentially, equivalent to a linear reduction in log antibody titre over time. Denote *A*_*ijk*_ to be the antibody titre to antigen *j* in participant *i* at time *t*_*k*_ which can therefore be described by the following linear model:

$$\begin{array}{l} \log\left(A_{ijk}\right)∼\left(\log\left(\alpha_{j}^{0}\right)+\log\left(\alpha_{ij}\right)\right)+\left(r_{j}^{0}+r_{ij}\right)t_{k}+\varepsilon_{j}\\ \log\left(\alpha_{ij}\right)∼N\left(0,\sigma_{A,j}\right)\\ \log\left(r_{ij}\right)∼N\left(0,\sigma_{r,j}\right)\\ \varepsilon_{j}∼N\left(0,\sigma_{m,j}\right) \end{array}$$

where $\alpha_{j}^{0}$ is the geometric mean titre (GMT) at the time of infection; log(*α*_*ij*_) is a random effect accounting for the difference between participant *i*'s initial antibody titre and the population-level GMT; $r_{j}^{0}$ is the average rate of decay of antigen *j* in the population; *r*_*ij*_ is a random effect for the difference between the decay rate of individual *j* with the population-level average; and *ε*_*j*_ is a Normally distributed error term.

The model was only fitted to individuals who were seropositive at baseline. This model also generated an estimate of the total variation in the data (arising from initial antibody level measured, the rate of antibody decay and the measurement error). Correlations between the three cohorts were performed using the Spearman’s rank correlation coefficient.

## Results

### Volunteer characteristics

Plasma samples were utilised from three studies in different geographic regions, with slightly different study designs. PNG included only children aged 5–10 years, whilst Brazil and Thailand included volunteers of all ages. Furthermore, all Thai and Brazilian volunteers were enrolled following symptomatic infection, whilst all included PNG children had asymptomatic infections. This resulted in significantly higher antigenic inputs in the Thai and Brazilian volunteers compared to PNG children (p<0.0001, Kruskal-Wallis test with Dunn’s multiple comparisons test) (Table 1).

**Table 1:** Volunteer characteristics.

	Brazil (*n* = 33)	Thailand (*n* = 32)	PNG (*n* = 31)
*Epidemiologic overview*			
Age, average (range)	36 years (16–56)	29 years (7–71)	8 years (5–10)
Sex, percentage male	79%	56%	55%
qPCR *Pv* prevalence (population-level)	5%	11.4%	47%
*Study design*			
Data collected	Apr 2014 –Sep 2015 (symptomatic)	May 2014 –May 2015 (symptomatic)	Aug 2009 –May 2010 (asymptomatic)
Treatment	DOT CQ (3 days) + 15 mg PQ (7 days)	DOT CQ (3 days) + 15 mg PQ (14 days)	DOT CQ + AL (3 days) + 0.5 mg/kg PQ (20 days)
Samples used	0, 3, 6, 9 month plasma	0, 3, 6, 9 month plasma	0, 3, 5 month plasma
*Results overview*			
Mean breadth* +ve antigen per person	160/307	211/307	175/307
Mean breadth* +ve persons per antigen	17/33	22/32	18/31
Geometric mean titre* median (range)	0.02 (8.3x10^-5^, 299)	0.18 (1.8x10^-4^, 202)	0.22 (1.7x10^-5^, 417)
Proportion of short-lived antibodies (half-life < 6 months)	88%	79%	59%
QMAL copies/μL (range)	1.18x10^5^ (229–5.12x10^5^)	1.62x10^6^ (4.93x10^4^-6.69x10^6^)	10 (0.002–83)

*Measured at first time-point. QMAL performed as previously described [41].

### Comparison of seropositivity to 307 *P*. *vivax* proteins between three different geographic regions

Reactivity was defined as more than 10% of the volunteers above the seropositivity cut-off, with the results at baseline (time of *P*. *vivax* infection) used for this analysis. Of the 307 *P*. *vivax* proteins assessed, 302 were considered reactive in Thailand, 273 in Brazil and 236 in PNG, with the population level breadth therefore highest in Thailand. All proteins considered reactive in Brazil and PNG were also considered reactive in Thailand. There were 3 proteins considered reactive in PNG and not Brazil (all with low levels of seropositivity, less than 30%), 29 proteins reactive in Thailand and not Brazil (seropositivity less than 35%), 40 proteins reactive in Brazil and not PNG (less than 50% seropositivity), and 65 proteins reactive in Thailand and not PNG. The majority of the reactive proteins identified in Thailand that were not observed in PNG had less than 50% seropositivity at the time of infection, however there were 6 exceptions: PVX_082690 (MSP7), PVX_092995 (Pv-fam-a), PVX_079980 (hypothetical protein), PVX_110945 (hypothetical protein) and PVX_099900 (unspecified product).

**Fig 2:**
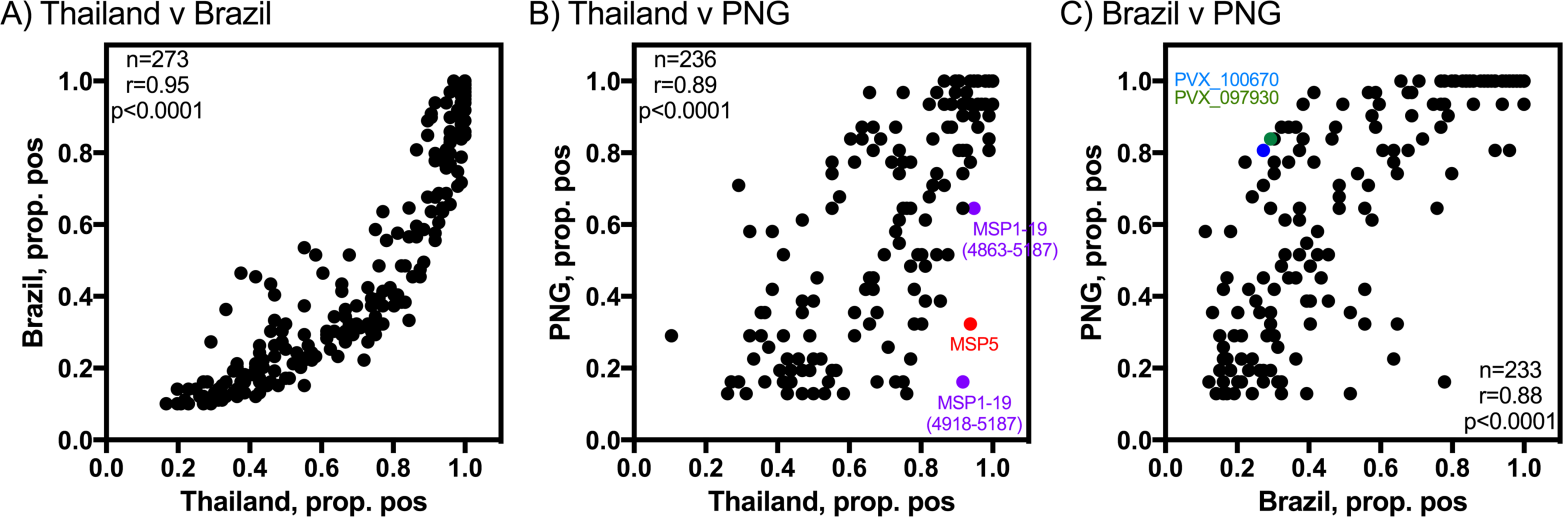
Correlation between seropositivity rates for antigens identified as reactive in multiple cohorts. (A) Thailand v Brazil, (B) Thailand v PNG and (C) Brazil v PNG. Spearman correlation coefficients, r, are shown. A number of proteins with highly different seropositivity rates between two sites are marked.

Overall, there was a strong correlation between seropositivity rates at baseline for the overlapping reactive antigens between the three cohorts (Thailand v Brazil, r = 0.95; Thailand v PNG, r = 0.89; Brazil v PNG, r = 0.88; all p<0.0001) (Fig 2). There were two outliers when comparing Thailand and PNG: MSP1-19 (PVX_099980, fragment covering 4918–5187 base pairs) and MSP5 (PVX_003770) (Fig 2B). Both these proteins induced high levels of seropositivity at the time of *P*. *vivax* infection in Thailand (92% and 94%, respectively), but low levels in PNG (16% and 32%, respectively). We also measured IgG responses against another construct of MSP1-19, base pairs 4863–5187, and responses were similarly higher in Thailand (95%) compared to PNG (65%), although the difference was not as large. There were also two proteins where seropositivity levels were high in PNG (more than 80%), but relatively low in Brazil (less than 30%): PVX_100670 (aspartyl protease, putative) and PVX_097930 (a hypothetical protein) (Fig 2C).

**Fig 3:**
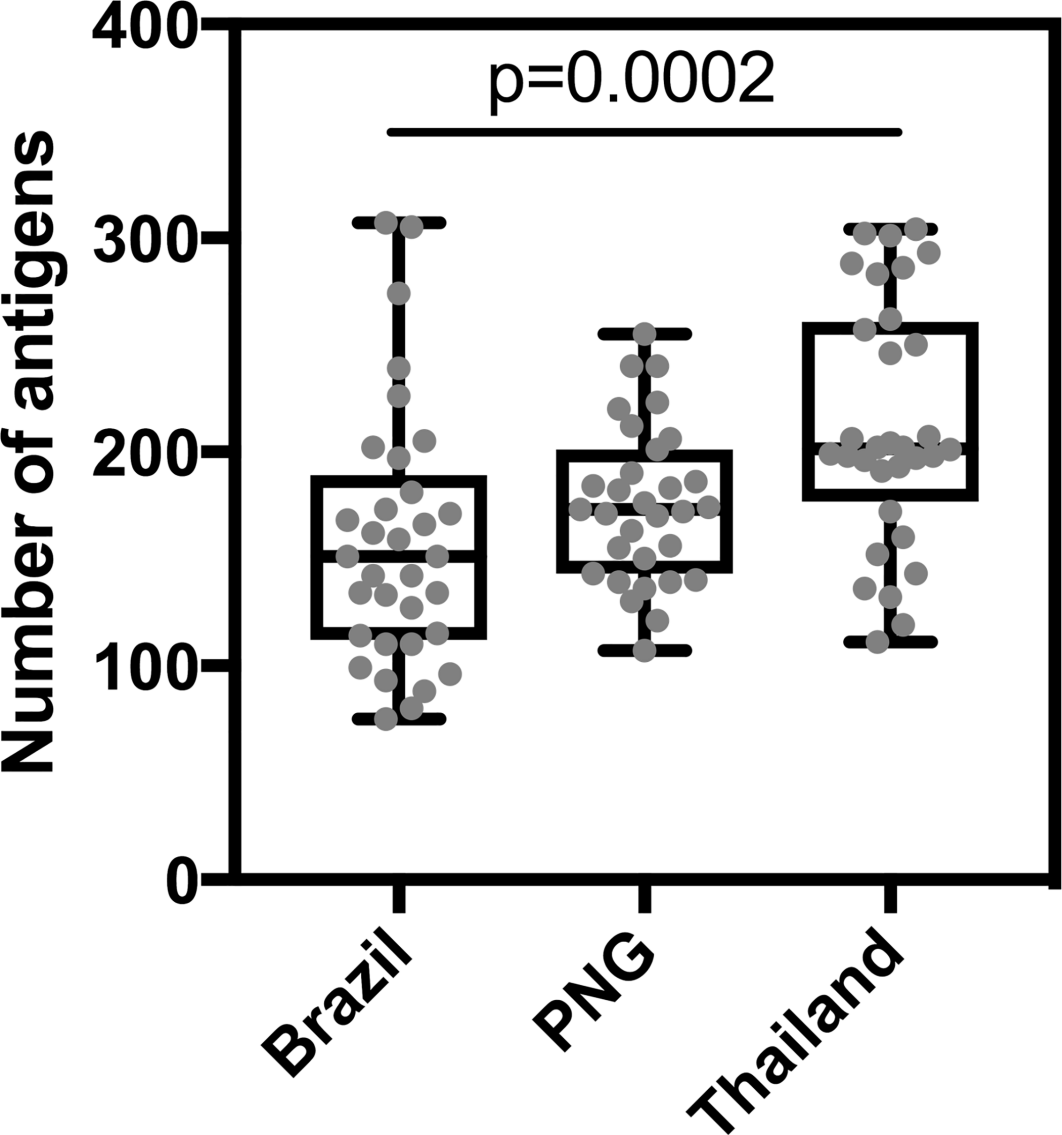
Breadth of the antibody response per person per cohort, at the time of *P*. *vivax* infection. The number of antigens each individual was seropositive for is shown, with box-plots indicating the median and ranges for each cohort site. Statistical difference was assessed using the Kruskal-Wallis test (p = 0.0004); after adjustment for multiple comparisons with Dunn’s test, p = 0.0002 between Brazil and Thailand only.

On an individual level, the breadth of the antibody response (that is, the number of antigens an individual was seropositive for) was slightly higher in Thailand and PNG compared to Brazil, at the time of *P*. *vivax* infection (Fig 3). The median number of antigens an individual was seropositive for was 202 in Thailand, 173 in PNG and 151 in Brazil. There was a statistically significant difference between the three sites (p = 0.0004, Kruskal-Wallis test).

### Comparison of antibody magnitude to reactive proteins between three different geographic regions

**Fig 4:**
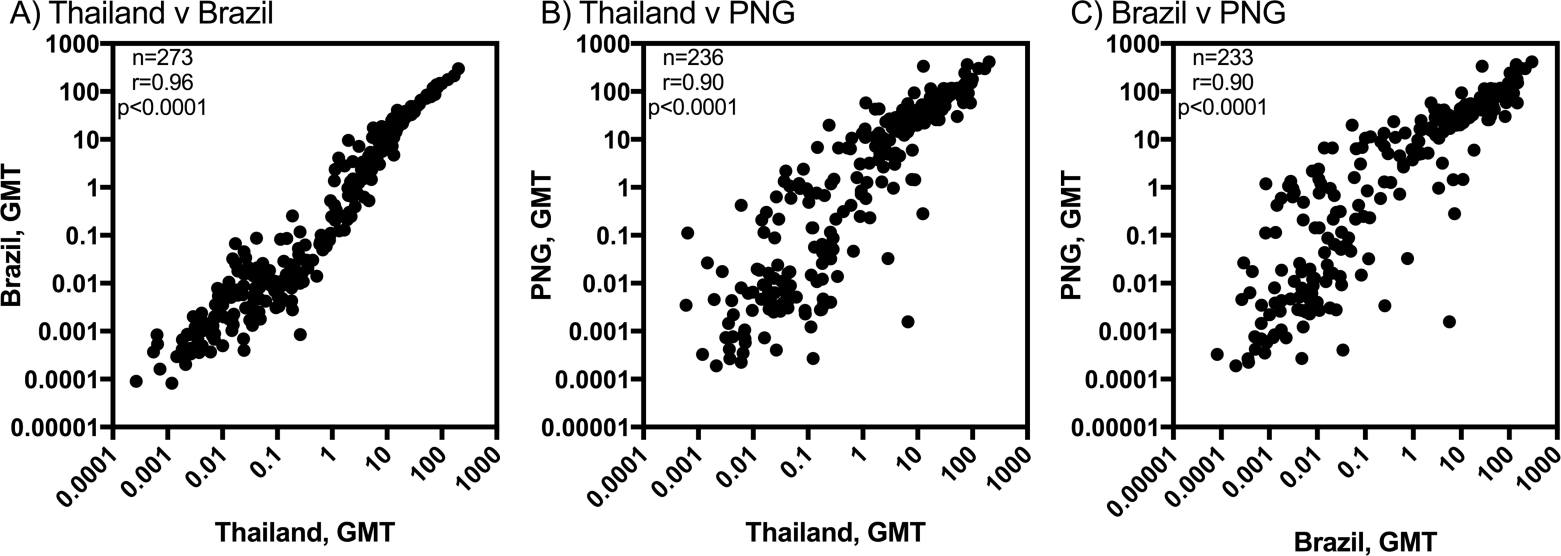
Correlation between the geometric mean antibody responses for antigens identified as reactive in multiple cohorts, at the time of *P*. *vivax* infection. (A) Thailand v Brazil, (B) Thailand v PNG and (C) Brazil v PNG. Spearman correlation coefficients, r, are shown.

In the AlphaScreen assay, the amount of protein used is not consistent between different proteins. Therefore, the absolute magnitude of the antibody response measured cannot be compared across proteins. However, the magnitude can be compared between individuals or populations for the same proteins. The geometric mean antibody level was determined for each protein for each study site, at the time of *P*. *vivax* infection. This value was then correlated between different sites; for the reactive antigens in common at baseline, the geometric mean was significantly correlated between all three cohorts (Thailand v Brazil, r = 0.96; Thailand v PNG, r = 0.90; Brazil v PNG, r = 0.90; all p<0.0001) (Fig 4). Overall, slightly higher mean antibody levels were measured in PNG compared to both Brazil and Thailand.

### Comparison of antibody longevity to reactive proteins in three different geographic regions

**Fig 5:**
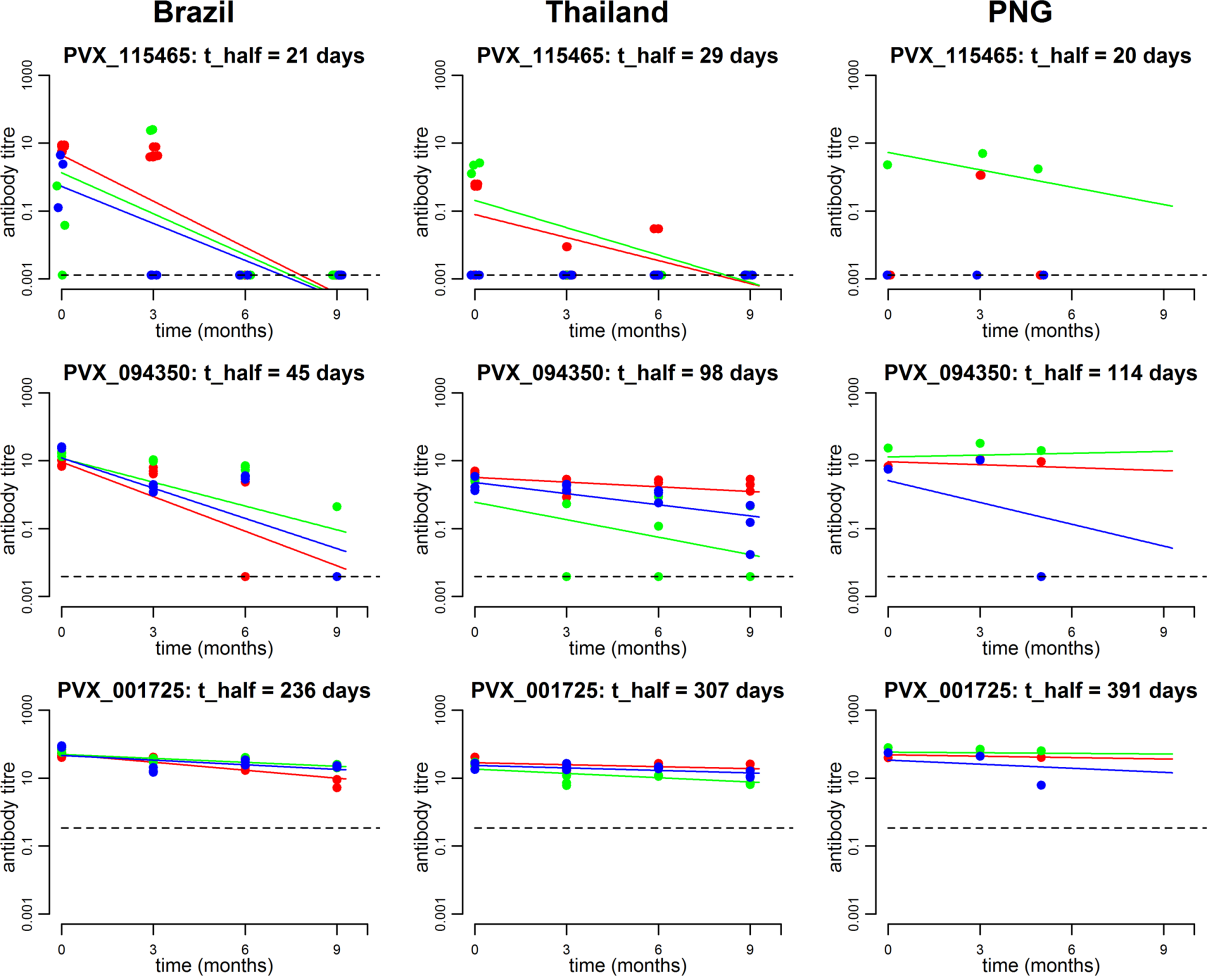
Example of antibody longevity to three different proteins for three randomly selected participants from each of the three study sites. The first row provides an example of a typical protein inducing a short-lived antibody kinetic profile; the middle row provides an example of a moderate-lived profile; the final row provides an example of an exceptionally long-lived profile. Each plot provides data from three individuals, who are differentially colour coded. The dashed line denotes the sero-positivity cut-off.

**Fig 6:**
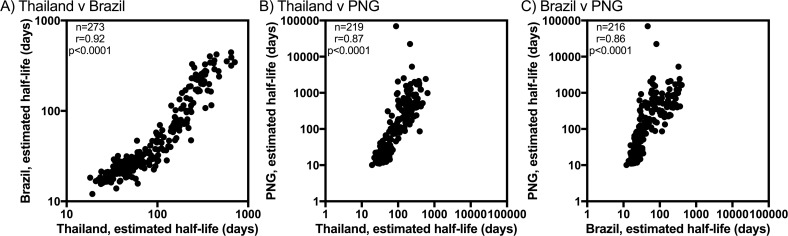
Correlation between the antibody longevity (estimated half-life) for antigens identified as reactive in multiple cohorts. Only antigens with positive half-life estimates were included. (A) Thailand v Brazil, (B) Thailand v PNG and (C) Brazil vs. PNG. Spearman correlation coefficients, r, are shown. Data are plotted on log scales to aid visualisation.

Antibody responses to the 307 *P*. *vivax* proteins were measured at multiple time-points; this made it possible to estimate the antibody longevity for the reactive proteins in each cohort (Fig 5). Antibody responses with an estimated half-life of less than 6 months (180 days) were considered short-lived, whereas those more than 6 months were considered long-lived. The majority of reactive antigens in all cohorts were considered short-lived, with the greatest proportion in Brazil (88%), followed by Thailand (79%), then PNG (59%). The range of estimated antibody half-lives in each cohort were as follows: 12–445 days in Brazil, 18–713 days in Thailand and 10–70,000 days in PNG. With 5 to 9 months of longitudinal data, there is limited statistical power to estimate very long half-lives, and hence a half-life of greater than 1,000 days is usually indistinguishable from no change in antibody titres over time. Overall, there was a strong correlation between estimated antibody longevity estimates for reactive proteins, when proteins with negative antibody half-life estimates were excluded (Thailand v Brazil, r = 0.92, n = 273 proteins; Thailand v PNG, r = 0.87, n = 219 proteins; Brazil v PNG, r = 0.86, n = 216 proteins; all p<0.0001) (Fig 6).

Concerning a number of well-recognised *P*. *vivax* antigens mentioned above, IgG responses to MSP1-19 and MSP5 were estimated to be exceptionally short-lived in all three cohorts, with half-lives of 16–41 days and 20–57 days, respectively. Responses to MSP1-42 were similarly short-lived in Brazil and Thailand (half-lives of 58–97 days) but were long-lived in PNG (estimated IgG half-life of almost 3 years).

## Discussion

In this study we have compared naturally acquired IgG antibody responses to over 300 *P*. *vivax* protein constructs in three geographically different locations with varying levels of *P*. *vivax* transmission. Manaus, Brazil and Tak, Thailand are both low-transmission regions (with approximately 0–5% and 1–11% of individuals infected in recent surveys, respectively [13,21, 31]). The participants from PNG were part of a larger study where a PCR prevalence of 47% was measured at baseline [20]. At the time of *P*. *vivax* infection we observed that a high proportion of the 307 *P*. *vivax* proteins were reactive (77–98%). This is higher than previous reports using large-scale screening platforms, where sero-reactivity has ranged from 14–50% in *P*. *vivax* patients [11, 17, 22], likely due both to our down-selection of proteins predicted to be immunogenic and our definition of seropositivity (based on the limit of detection of the assay, rather than unexposed controls). We observed higher numbers of sero-reactive proteins in Thailand and Brazil compared to PNG; the volunteers in Thailand and Brazil who had symptomatic infections also had significantly higher parasite densities than the asymptomatic children in PNG, signifying that antigenic input has a significant effect on the breadth of the antibody response. Similarly, on an individual level, Thai volunteers showed a trend towards a greater breadth of response compared to Brazil and PNG. This pattern of greater breadth of response in individuals with higher parasite densities [11], and following symptomatic versus asymptomatic infections [13], has been reported previously. As asymptomatic infections are becoming increasingly recognised in low-transmission regions such as Thailand [13], it would be valuable to compare the breadth of response at the time of asymptomatic *P*. *vivax* infection in Thailand, or a similar low-transmission region, to that in PNG.

The additional proteins identified as sero-reactive in Thailand and Brazil compared to PNG had only low levels of seropositivity amongst the 32–33 individuals at time of infection (i.e. between 10–50%), except for a small number of proteins in the Thai individuals including one Pv-fam-a protein (PVX_092995) and MSP7 (PVX_082690). In general, there was good correlation in levels of seropositivity to all antigens considered reactive in multiple cohorts. Two key exceptions were observed: high levels of seropositivity in Thai individuals to MSP1-19 (fragment covering base pairs 4918–5187) and MSP5 (92% and 94%, respectively), with only low-levels of seropositivity observed in the PNG children (16% and 32%, respectively). MSP1 and MSP5 are both blood-stage antigens considered potential vaccine candidates, based primarily on their location on the merozoite surface and pre-clinical studies of the *P*. *falciparum* orthologs [32, 33]. MSP1 first exists as a 42 kDa protein attached to the surface of merozoites via a GPI anchor; following invasion of RBCs the protein is cleaved into two products, of 33 kDa and 19 kDa, with the 19 kDa fragment remaining on the merozoite surface [34]. We observed 100% seropositivity in all cohorts to the MSP1-42 fragment. Not a lot is known about IgG responses to MSP5 [33], but responses to MSP1-19 are frequently observed in endemic regions [35], and are known to be short-lived [36]. We observed short-lived responses to both MSP1-19 and MSP5 in all three study sites. In agreement with our current finding, higher IgG titres to MSP1-19 have been observed in sporadically rather than chronically exposed volunteers in an endemic region of Brazil [37]; together this suggests that in chronically exposed individuals, such as PNG children, MSP1-19 and MSP5 may be able to escape recognition of the humoral immune system or that such low-density infections are unable to adequately boost the IgG response. However, it is important to note that we observed moderate levels of seropositivity in PNG children to a second construct of MSP1-19 (covering base pairs 4863–5187), and another study has also found prevalent IgG responses to MSP1-19 in younger PNG children [38]. Our current finding therefore warrants further investigation with a larger sample size to clarify these results.

The majority of previous studies that have assessed IgG antibody responses to a large panel of *P*. *vivax* proteins have been cross-sectional in design; whilst this provides useful information on antibody acquisition and immunogenicity, it fails to provide detailed information regarding antibody longevity. In this study we assessed antibody responses at 2–3 further time points following *P*. *vivax* infection, in the absence of detectable recurrent infections, to investigate antibody decay. We observed that in our two low-transmission study sites, Thailand and Brazil, most antigens had relatively short-lived IgG responses, with estimated antibody half-lives of less than 6 months. It is important to note that this was not observed for all proteins, with some exhibiting exceptionally long-lived IgG profiles with half-lives up to 700 days, and we are currently investigating the mechanisms behind such differential responses. Conversely, in the PNG children who had a high level of past, lifetime exposure [6], 41% of proteins induced long-lived IgG responses with estimated half-lives of more than 6 months, and there was a trend towards longer-lived responses to many proteins in these children compared to individuals from Thailand and Brazil. This suggests that whilst the breadth of response may be dependent upon the level of antigenic input, IgG responses can be long-lived to many proteins if there has been sufficient past levels of exposure within a region. Together, this provides further evidence that IgG responses to *P*. *vivax* proteins can be long-lived, dependent on the transmission setting and specific protein, which is in contrast to most reported studies of IgG longevity against *P*. *falciparum* proteins, where responses are generally short-lived in the absence of ongoing exposure [35, 39].

Despite these differences between study sites in terms of the breadth and longevity of the IgG response, there was a highly significant rank-correlation between sites in terms of the proportion of individuals seropositive to each antigen, the magnitude of the IgG response to each antigen, and the estimated antibody half-life to each antigen. This suggests that despite the differences due to parasite density and past exposure levels, immunogenicity and longevity are ultimately characteristics specific to individual proteins. This feature is therefore highly promising for the use of IgG antibody responses as markers of exposure in multiple geographic regions, if the transmission level and current infection status is taken into consideration. An important consideration for future research is the impact of age on the IgG responses observed; our current work has attributed differences in the PNG cohort to the higher level of transmission within this region and lower antigenic input due to asymptomatic infection, however the age of the volunteers could also have an effect. In addition, longitudinal studies of individuals from low-transmission regions following asymptomatic *P*. *vivax* infections would also be valuable to further support our hypotheses. Furthermore, the potential for cross-reactivity of these *P*. *vivax* proteins with *P*. *falciparum* will become increasingly important in regions where these species are co-endemic. The potential for cross-reactivity will be tested by measuring responses in cohorts which are known to have *P*. *falciparum* but no *P*. *vivax* [40]. In summary, our study has utilised a panel of more than 300 *P*. *vivax* proteins, the majority of which were immunogenic, to provide evidence for a number of key features relating to IgG acquisition and longevity: that the individual and population-level breadth of IgG seropositivity is a function of antigenic input, whilst longevity is a function of the level of transmission (and hence lifetime exposure to the parasite).
